# Biomarker of exposure level data set in smokers switching from conventional cigarettes to Tobacco Heating System 2.2, continuing smoking or abstaining from smoking for 5 days

**DOI:** 10.1016/j.dib.2016.11.047

**Published:** 2016-11-18

**Authors:** Christelle Haziza, Guillaume de La Bourdonnaye, Dimitra Skiada, Jacek Ancerewicz, Gizelle Baker, Patrick Picavet, Frank Lüdicke

**Affiliations:** PMI R&D, Philip Morris Products S.A (Part of Philip Morris International Group of Companies), Neuchâtel, Switzerland

**Keywords:** Reduced risk product, Tobacco exposure, Clinical study, Tobacco heating system 2.2

## Abstract

Levels of biomarkers of exposure to selected harmful and potentially harmful smoke constituents found in cigarette smoke, in addition to nicotine were measured in 160 smokers randomized for 5 days to continuing smoking conventional cigarettes (41 participants), switching to Tobacco Heating System 2.2 (THS 2.2) (80 participants), or abstaining from smoking (39 participants). The data reported here are descriptive statistics of the levels of each biomarker of exposure expressed as concentrations adjusted to creatinine; at baseline, and at the end of the study, and their relative change from baseline. Reductions in the levels of biomarkers of exposure when expressed as quantity excreted, are also reported. Detailed descriptions of bioanalytical assays used are also provided. The data presented here are related to the article entitled “Evaluation of the Tobacco Heating System 2.2. Part 8: 5-Day randomized reduced exposure clinical study in Poland” (Haziza et al., 2016) [1].

**Specifications Table**

**Table:** 

Subject area	Pharmacology, toxicology, and pharmaceutical science
More specific subject area	Tobacco Harm reduction
Type of data	*Table, text*
How data was acquired	Liquid chromatography-tandem mass spectrometry, spectrophotometry, and gas chromatography–mass spectrometry.
Data format	*Analyzed*
Experimental factors	*Urine and blood sampling collected from healthy smokers participating in the clinical study. This study is disclosed on clinicaltrials.gov (*NCT01959932*)*
Experimental features	Measurements of biomarkers of exposure to selected harmful and potentially harmful constituents found in cigarette smoke and to to nicotine y after 5 days of use of THS 2.2, conventional cigarettes, or smoking abstinence.
Data source location	*Warsaw, Poland*
Data accessibility	*Data are within this article.*

**Value of the data**

• The product tested in this clinical study, the Tobacco Heating System 2.2 (THS 2.2), was developed by Philip Morris International (PMI) as a candidate reduced-risk product aiming to provide an acceptable alternative to CC smoking, replicating the ritual, taste, sensory characteristics, and nicotine uptake of conventional cigarette smoking.
• The data provide an understanding on how the levels of biomarkers of exposure compare in between smokers switching to THS 2.2 or smoking conventional cigarettes.
• The data may be of value to the scientific community because it offers further insights to other researchers in the context of tobacco harm reduction strategies.

## 1 Data

Table 1 describes the levels of 15 biomarkers of exposure to selected harmful and potentially harmful smoke constituents, along with the levels of biomarkers of exposure to nicotine, measured at baseline and after 5 days of the clinical study investigational period. Carboxyhemoglobin was measured in in blood (expressed as % of saturation of hemoglobin), nicotine and cotinine were measured in plasma (expressed in ng/mL), while the other biomarkers of exposure were measured in 24-hour urine (expressed as concentration adjusted to creatinine). Table 2 shows % of change from baseline within groups for each biomarker of exposure. Inferential analysis testing (ratio THS/CC expressed as %) using an ANCOVA model with adjustment to baseline values, sex, conventional cigarette consumption reported at screening, and study arms, is presented in Table 3.

## 2 Experimental design, materials and methods

This study was designed as a controlled, randomized, three-arm parallel, single-center study in confinement [1]. After the screening visit, the study included 1 day for admission, 2 days of baseline where smokers were smoking their own brand of CC, followed by a 5 day randomized investigational period distributed as follows: 41 subjects were randomized to continue CC smoking, 39 subjects were randomized to smoking abstinence, and 80 subjects were randomized to switch from CC to THS 2.2. At baseline and 5 days of the investigational period, blood and 24-hour urine were collected to measure 15 biomarkers of exposure to selected HPHCs. In addition, biomarkers of exposure to nicotine were assessed.

Bioanalytical work was performed by Celerion Laboratories at Celerion USA, Lincoln, NE, United States. The assays used for bioanalytical analysis and described below were implemented and validated in accordance with Celerion standard operating procedures which were written to meet the FDA Guidance to Industry [2].

## 3 Nicotine equivalents in urine

Bioanalytical assays were developed to quantitate nicotine, cotinine, *trans*-3′-hydroxycotinine, nicotine-*N*-glucuronide, cotinine-*N*-glucuronide, and *trans*-3′-hydroxycotinine-*O*-glucuronide in human urine. To reduce the number of reassays and sample dilutions required to achieve a measurable result, two linear ranges were validated to quantitate samples from subjects that receive different treatments. For subjects that were randomized to smoking cessation a nine point standard calibrator line was established across the nicotine (10.0–1000 ng/mL), cotinine (10.0–1000 ng/mL), *trans*-3′-hydroxycotinine (10.0–1000 ng/mL), nicotine-*N*-glucuronide (10.0–1000 ng/mL), cotinine-*N*-glucuronide (20.0–2000 ng/mL), and *trans*-3′-hydroxycotinine-*O*-glucuronide (50.0–5000 ng/mL) analytical ranges. For subjects randomized to conventional cigarettes or the test product the linear ranges of nicotine (50.0–5000 ng/mL), cotinine (50.0–5000 ng/mL), *trans*-3′-hydroxycotinine (50.0–5000 ng/mL), nicotine-*N*-glucuronide (50.0–5000 ng/mL), cotinine-*N*-glucuronide (200–20,000 ng/mL), and *trans*-3′-hydroxycotinine-*O*-glucuronide (200–20,000 ng/mL) were validated. Calibration standards, quality control samples and clinical samples were supplemented with stable label internal standards (d_4_-nicotine, d_3_-cotinine, d_3_-*trans*-3′-hydroxycotinine, d_3_-nicotine-*N*-glucuronide, d_3_-cotinine-*N*-glucuronide, d_3_-*trans*-3′-hydroxycotinine-*O*-glucuronide). The target analytes were retained through combined liquid-liquid and solid-phase extraction processes. The eluents were dried under a stream of nitrogen gas. The extracts were reconstituted with deionized water and were injected onto an LC-MS/MS system for detection. Positive ions were monitored in multiple reaction monitoring mode.

The validation testing of the lower concentration range included precision and accuracy testing with 6 replicates of quality control samples prepared at 4 concentrations (nicotine <9.3% C.V., <7.0% bias; cotinine <8.6% C.V., <10.6% bias; *trans*-3′-hydroxycotinine <11.5% C.V., <9.5% bias; nicotine-*N*-glucuronide <9.2% C.V., <11.3% bias; cotinine-*N*-glucuronide <11.3% C.V., <10.2% bias; *trans*-3′-hydroxycotinine-*O*-glucuronide <8.9% C.V., <7.8% bias), recovery (nicotine 90–101%; cotinine 91–100%; *trans*-3′-hydroxycotinine 49–52%; nicotine-*N*-glucuronide 47–54%; cotinine-*N*-glucuronide 46–51%; *trans*-3′-hydroxycotinine-*O*-glucuronide 42–49%), multiple-lot quantitation, stock/sub-stock solution stability at −20 °C in polypropylene containers, long-term clinical sample stability stored at −20 °C (440 days), freeze/thaw stability (6 cycles under white light), short-term stability (54 h under white light at ambient temperature), and post-preparative stability (180 h). The validation testing of the higher concentration range included precision and accuracy testing with 6 replicates of quality control samples prepared at 4 concentrations (nicotine <9.9% C.V., <13.3% bias; cotinine <5.8% C.V., <12.8% bias; *trans*-3′-hydroxycotinine <7.5% C.V., <12.3% bias; nicotine-*N*-glucuronide <7.6% C.V., <3.8% bias; cotinine-*N*-glucuronide <9.8% C.V., <11.5% bias; *trans*-3′-hydroxycotinine-*O*-glucuronide <8.3% C.V., <3.0% bias), recovery (nicotine 100–106%; cotinine 109–120%; *trans*-3′-hydroxycotinine 49–53%; nicotine-*N*-glucuronide 48–51%; cotinine-*N*-glucuronide 44–52%; *trans*-3′-hydroxycotinine-*O*-glucuronide 39–46%), multiple-lot quantitation, stock/sub-stock solution stability at −20 °C in polypropylene containers, long-term clinical sample stability stored at −20 °C (476 days), freeze/thaw stability (6 cycles under white light), short-term stability (57 h under white light at ambient temperature), and post-preparative stability (229 h). All data from the method validations were reviewed by an independent quality assurance unit as per Celerion standard operating procedures.

The validated assay was successfully employed to measure the clinical samples with duplicate quality controls prepared at 4 concentrations.

## 4 Nicotine, and cotinine in plasma

For the analysis of nicotine, cotinine in human EDTA plasma 3 bioanalytical assays were validated. A validated lower concentration range assay was utilized for subjects that were randomized to smoking cessation. A nine point standard calibrator line was established across the nicotine (0.200–10.0 ng/mL), and cotinine (1.00–100 ng/mL) analytical ranges. Calibration standards, quality control samples and clinical samples were supplemented with stable label internal standards (d_4_-nicotine, d_3_-cotinine). The target analytes were retained through a 96-well solid-phase extraction process. The eluent was injected after dilution onto an LC-MS/MS system for detection. Positive ions were monitored in multiple reaction monitoring mode.

The validation testing included precision and accuracy testing with 6 replicates of quality control samples prepared at 4 concentrations (nicotine <6.2% C.V., <5.5% bias; cotinine <2.2% C.V., <4.2% bias), recovery (nicotine 94–97%, cotinine 75–83%), multiple-lot quantitation, hemolyzed sample integrity, turbid sample integrity, aqueous stock/sub-stock solution stability at −20 °C in polypropylene, long-term clinical sample stability (nicotine 1041 days, cotinine 739 days), freeze/thaw stability (6 cycles under white light), short-term stability (27 h under white light at ambient temperature), and post-preparative stability (321 h). All data from the method validation was reviewed by an independent quality assurance unit as per Celerion standard operating procedures.

Two higher concentration range assays were validated and then utilized for the analysis of clinical samples from subjects that were randomized to conventional cigarette or test product use. for the study, a nine point standard calibrator line was established across the nicotine (1.00–50.0 ng/mL), and cotinine (1.00–100 ng/mL) analytical ranges. Calibration standards, quality control samples and clinical samples were supplemented with stable label internal standards (d_4_-nicotine, d_3_-cotinine). The target analytes were retained through a 96-well solid-phase extraction process. The eluent was injected after dilution onto an LC-MS/MS system for detection. Positive ions were monitored in multiple reaction monitoring mode.

The validation testing for both high range assays included precision and accuracy testing with 6 replicates of quality control samples prepared at 4 concentrations (nicotine <7.4% C.V., <7.0% bias; cotinine <11.3% C.V., <10.0% bias), recovery (nicotine 72–78%, cotinine 81–86%), multiple-lot quantitation, hemolyzed sample integrity, turbid sample integrity, aqueous stock/sub-stock solution stability at −20 °C in polypropylene, long-term clinical sample stability (220 days), freeze/thaw stability (6 cycles under white light), short-term stability (49 h under white light at ambient temperature), and post-preparative stability (142 h). All data from the method validation was reviewed by an independent quality assurance unit as per Celerion standard operating procedures.

The validated assay was successfully employed to measure the clinical samples with duplicate quality controls prepared at 3 concentrations.

## 5 3-HPMA, HBMA, and CEMA in urine

A bioanalytical assay developed to quantitate 3-Hydroxypropyl Mercapturic Acid (3-HPMA), Hydroxybutyl Mercapturic Acid (HBMA) and Cyanoethylmercapturic Acid (CEMA) in human urine. A 10 point standard calibrator line was established in artificial urine (CST Technologies) across the 3-HPMA and HBMA (20.0–5000 ng/mL) and CEMA (0.275–207 ng/mL) analytical ranges. Calibration standards, quality control samples and clinical samples were supplemented with stable label internal standards (^15^N^13^C_3_-3-HPMA, ^15^N^13^C_3_-HBMA, and ^15^N^13^C_3_-CEMA). The target analytes were retained through a 96-well solid-phase extraction process. The eluents were dried under a stream of nitrogen gas. The xtracts were reconstituted in deionized water and were injected onto an LC-MS/MS system for detection. Negative ions were monitored in multiple reaction monitoring (MRM) mode.

The validation testing included precision and accuracy testing with 6 replicates of quality control samples prepared at 6 concentrations (3-HPMA <10.2% C.V., <6.3% bias; HBMA <7.6 C.V., 13.5% bias, CEMA <10.0% C.V., <4.4% bias), recovery (3-HPMA 54–64%, HBMA 76–83%, CEMA 82–86%), multiple-lot quantitation, stock/sub-stock solution stability at −20 °C in polypropylene, long-term clinical sample stability (273 days at −20 °C), freeze/thaw stability (7 cycles in brown tubes), short-term stability (43 h in brown tubes at ambient temperature), and post-preparative stability (152 h in a polypropylene 96 well plate at 5 °C). All data from the method validation was reviewed by an independent quality assurance unit as per Celerion standard operating procedures.

The validated assay was successfully employed to measure the clinical samples with duplicate quality controls prepared at 5 concentrations.

## 6 Aromatic amines in urine

A bioanalytical assay developed to quantitate *o*-toluidine (o-tol), 1-aminonaphthalene (1-NA), 2-aminonaphthalene (2-NA) and 4-aminobiphenyl (4-ABP) in human urine. An eight point standard calibrator line was established in artificial urine (CST Technologies) across the *o*-toluidine (50.0–1000 pg/mL), 1-aminonaphthalene and 2-aminonaphthalene (2.50–500 pg/mL), and 4-aminobiphenyl (1.50–75.0 pg/mL) analytical ranges. Calibration standards, quality control samples and clinical samples were supplemented with stable label internal standards ([^13^C_6_]*o*-toluidine, [^13^C_4_]2-aminonaphthalene, [^13^C_6_]4-aminobiphenyl). The target analytes were retained through a liquid/liquid extraction process. The extraction solvent was dried under a stream of nitrogen gas. The extracts were reconstituted in a polar organic solvent and injected onto an LC-MS/MS system for detection. Positive ions were monitored in multiple reaction monitoring (MRM) mode.

The validation testing included precision and accuracy testing with 6 replicates of quality control samples prepared at 5 concentrations (*o*-toluidine <9.9% C.V., <3.7% bias; 1-NA <11.7% C.V., <3.3% bias; 2-NA <12.0 C.V., <5.6% bias, 4-ABP <5.2% C.V., <8.1% bias), recovery (*o*-toluidine 80.0%, 1-NA 53.0–55.0%, 2-NA 81.0–84.0%, 4-ABP 82.0–84.0%), multiple-lot quantitation, stock/sub-stock solution stability at −20 °C in polypropylene, long-term clinical sample stability (185 days at −20 °C), freeze/thaw stability (6 cycles at ambient temperature in brown polypropylene tubes), short-term stability (54 h at ambient temperature), and post-preparative stability (337 h). All data from the method validation was reviewed by an independent quality assurance unit as per Celerion standard operating procedures.

The validated assay was successfully employed to measure the clinical samples with duplicate quality controls prepared at 4 concentrations.

## 7 MHBMA in urine

A bioanalytical assay was developed to quantitate monohydroxybutenyl mercapturic acid (MHBMA) in human urine. A ten point standard calibrator line was established in an artificial urine across the MHBMA (0.100–20.0 ng/mL) analytical range. Calibration standards, quality control samples and clinical samples were supplemented with stable label internal standards (^15^N^13^C_3_-3-MHBMA). The target analytes were retained through a solid-phase extraction process. The eluents were dried under a stream of nitrogen gas. The extracts were reconstituted in a polar organic solvent and injected onto an LC-MS/MS system for detection. Negative ions were monitored in multiple reaction monitoring mode.

The validation testing included precision and accuracy testing with 6 replicates of quality control samples prepared at 4 concentrations (<14.7% C.V., <6.0% bias), recovery (82–100%), multiple-lot quantitation, stock/sub-stock solution stability at −20 °C in polypropylene, long-term clinical sample stability (158 days at −20 °C), freeze/thaw stability (4 cycles in brown tubes), short-term stability (20 h protected from white light at ambient temperature), and post-preparative stability (112 h). All data from the method validation was reviewed by an independent quality assurance unit as per Celerion standard operating procedures.

The validated assay was successfully employed to measure the clinical samples with duplicate quality controls prepared at 4 concentrations.

## 8 S-PMA and S-BMA in urine

A bioanalytical assay was developed to quantitate *S*-phenyl mercapturic acid (S-PMA) and *S*-benzyl mercapturic acid (S-BMA) in human urine. A 10 point standard calibrator line was established in deionized water across the SPMA (25.0–6250 ng/mL) and S-BMA (100–25,000 pg/mL) analytical ranges. Calibration standards, quality control samples and clinical samples were supplemented with stable label internal standards (^13^C_3_^15^N-*S*-phenyl mercapturic acid, and ^13^C_3_^15^N-*S*-benzyl mercapturic acid). The target analytes were retained through a solid-phase extraction process. The eluents were dried under a stream of nitrogen gas. The extracts were reconstituted in a polar organic solvent and injected onto an LC-MS/MS system for detection. Negative ions were monitored in multiple reaction monitoring mode.

The validation testing included precision and accuracy testing with 6 replicates of quality control samples prepared at 4 concentrations (S-PMA <12.9% C.V., <14.0% bias, S-BMA <6.8% C.V., <8.0% bias), recovery (S-PMA 65–75%; S-BMA 79–84%), multiple-lot quantitation, stock/sub-stock solution stability at −20 °C in polypropylene, long-term clinical sample stability (161 days at −20 °C), freeze/thaw stability (4 cycles at ambient temperatures protected from light), short-term stability (81 hours at 5 °C protected from light), and post-preparative stability (181 hours). All data from the method validation was reviewed by an independent quality assurance unit as per Celerion standard operating procedures.

The validated assay was successfully employed to measure the clinical samples with duplicate quality controls prepared at 4 concentrations.

## 9 Total 1-OHP in urine

A bioanalytical assay was developed to quantitate total 1-hydroxypyrene (1-OHP) in human urine. An eight point standard calibrator line was established in artificial urine (CST Technologies) across the 1-OHP (10.0–2000 pg/mL) analytical range. Calibration standards, quality control samples and clinical samples were supplemented with stable label internal standard (^13^C_6_-1-OHP). All samples were treated with beta-glucuronidase to convert the glucuronide metabolites to the aglycone which was measured. The target analytes were retained through a solid-phase extraction process. The eluents were dried under a stream of nitrogen gas. The extracts were reconstituted with a polar solvent mixture and were injected onto an LC-MS/MS system for detection. Positive ions were monitored in multiple reaction monitoring mode.

The validation testing included precision and accuracy testing with 6 replicates of quality control samples prepared at 4 concentrations (<11.0% C.V., <13.4% bias), recovery (78.7–86.9%), multiple-lot quantitation, stock/sub-stock solution stability at −20 °C in polypropylene, long-term clinical sample stability (237 days), freeze/thaw stability (6 cycles under white light), short-term stability (17 h at ambient temperature), and post-preparative stability (55 h). All data from the method validation was reviewed by an independent quality assurance unit as per Celerion standard operating procedures.

The validated assay was successfully employed to measure the clinical samples with duplicate quality controls prepared at 4 concentrations.

## 10 Total NNN and total NNAL in urine

A bioanalytical assay was developed to quantitate NNAL and NNN in human urine. A 9 point standard calibrator line was established in ultrapure water across the NNAL (5.00–1000 pg/mL) and NNN (0.200–40.0 pg/mL) analytical ranges. Calibration standards, quality control samples and clinical samples were supplemented with stable label internal standards (d_4_-NNAL and d_4_-NNN). The target analytes were retained through a solid phase extraction process. The extraction solvent was dried under a stream of nitrogen gas. The extracts were reconstituted in a polar organic solvent and injected onto an LC-MS/MS system for detection. Positive ions were monitored in multiple reaction monitoring mode.

The validation testing included precision and accuracy testing with 6 replicates of quality control samples prepared at 4 concentrations (NNAL <18.2% C.V., <3.0% bias; NNN <24.6% C.V., <3.5% bias), recovery (NNAL 88.0–85.0%, NNN 64.0–66.0%), multiple-lot quantitation, stock/sub-stock solution stability at −20 °C in polypropylene, long-term clinical sample stability (807 days at −20 °C; 318 days (LLOQ QC) and 296 days (dilution QC) at −20 °C), freeze/thaw stability (6 cycles at ambient temperature for NNAL, 6 cycles at ambient temperature for NNN), short-term stability (24 h at ambient temperature for NNAL, 24 h at ambient temperature for NNN), and post-preparative stability (126 h for NNAL, 179 h for NNN). All data from the method validation was reviewed by an independent quality assurance unit as per Celerion standard operating procedures.

The validated assay was successfully employed to measure the clinical samples with duplicate quality controls prepared at 4 concentrations.

## 11 Total 3-hydroxy benzo[a]pyrene in urine

A bioanalytical assay was developed to quantitate 3-hydroxybenzo[a]pyrene in human urine. An eight point standard calibrator line was established across the 3-hydroxybenzo[a]pyrene (25.0–600 fg/mL) analytical range and was validated. Calibration standards, quality control samples and clinical samples were supplemented with stable label internal standard (^13^C_6_-3-hydroxybenzo[a]pyrene). All samples were treated with β-glucuronidase to convert the glucuronide metabolite to the aglycone which was measured. The target analyte was retained through a solid-phase extraction process. The eluents were dried under a stream of nitrogen gas. The extracts were reconstituted with a polar organic solvent and were injected onto an LC-MS/MS system for detection. Positive ions were monitored in multiple reaction monitoring mode.

The validation testing included precision and accuracy testing with 6 replicates of quality control samples prepared at 5 concentrations (<15.8% C.V., <2.8% bias), recovery (91–104%), multiple-lot quantitation, stock/sub-stock solution stability at −20 °C in silanized glass containers, long-term clinical sample stability stored at −20 °C (570 days), freeze/thaw stability (6 cycles under UV-shielded light), short-term stability (55 hours under UV-shielded at ambient temperature), and post-preparative stability (156 h). All data from the method validations were reviewed by an independent quality assurance unit as per Celerion standard operating procedures.

The validated assay was successfully employed to measure the clinical samples with duplicate quality controls prepared at 4 concentrations.

## 12 Caffeine and paraxanthine in plasma

A bioanalytical assay was developed to quantitate caffeine and paraxanthine in human plasma. A 9 point standard calibrator line was established in human plasma (heparin) across the caffeine and paraxanthine (20.0–5000 ng/mL) analytical ranges. Calibration standards, quality control samples and clinical samples were supplemented with stable label internal standards (d_9_-caffeine and d_3_-paraxanthine). The target analytes were retained through a liquid-liquid extraction process. The extraction solvent was dried under a stream of nitrogen gas. The extracts were reconstituted in a polar organic solvent and injected onto an LC-MS/MS system for detection. Positive ions were monitored in multiple reaction monitoring mode.

The validation testing included precision and accuracy testing with 6 replicates of quality control samples prepared at 4 concentrations (caffeine <7.9% C.V., <3.6% bias; paraxanthine <6.5% C.V., <−0.6% bias), recovery (caffeine 81.0–87.0%, paraxanthine 59.0–63.0%), multiple-lot quantitation, stock/sub-stock solution stability at −20 °C in polypropylene, long-term clinical sample stability (545 days at −20 °C), freeze/thaw stability (6 cycles at ambient temperature and in an ice water bath), short-term stability (24 h at ambient temperature and in an ice water bath), and post-preparative stability (162 h). All data from the method validation was reviewed by an independent quality assurance unit as per Celerion standard operating procedures.

The validated assay was successfully employed to measure the clinical samples with duplicate quality controls prepared at 4 concentrations.

## 13 HEMA in urine

A bioanalytical assay was developed to quantitate HEMA in human urine. A nine point standard calibrator line was established in artificial urine across the HEMA (0.100–20.0 ng/mL) analytical range. Calibration standards, quality control samples and clinical samples were supplemented with stable label internal standard (^15^N^13^C_3_-HEMA). The target analyte was retained through a solid phase extraction process. The extraction solvent was dried under a stream of nitrogen gas. The extracts were reconstituted in a polar organic solvent and injected onto an LC-MS/MS system for detection. Positive ions were monitored in multiple reaction monitoring mode.

The validation testing included precision and accuracy testing with 6 replicates of quality control samples prepared at 3 concentrations (<6.1% C.V., <4.9% bias), recovery (64–79%), multiple-lot quantitation, stock/sub-stock solution stability at −20 °C in polypropylene, long-term clinical sample stability (406 days at −20 °C), freeze/thaw stability (6 cycles at ambient temperature), short-term stability (27 h at ambient temperature), and post-preparative stability (205 h). All data from the method validation was reviewed by an independent quality assurance unit as per Celerion standard operating procedures.

The validated assay was successfully employed to measure the clinical samples with duplicate quality controls prepared at 4 concentrations.

## Figures and Tables

**Table 1:** Biomarkers of exposure levels during the study.

Biomarker	THS	CC	SA
	*N*=80	*N*=41	*N*=39
**NEQ**^a^**(mg/g creat)**			
Baseline	9.01 (8.09; 10.03)	8.69 (7.51; 10.04)	9.53 (8.44; 10.76)
Day 5	10.60 (9.34; 12.04)	9.76 (8.54; 11.15)	0.14 (0.12; 0.17)
**Nicotine**^a^, ^b^**(ng/mL)**			
Baseline	14.16 (12.62;15.89)	14.03 (11.90; 16.53)	14.39 (12.63; 16.40)
Day 5	20.74 (17.46; 24.62)	19.01 (16.52; 21.87)	0.10 (0.09; 0.12)
**Cotinine**^a^, ^b^**(ng/mL)**			
Baseline	208.54 (188.61; 230.58)	211.26 (183.05; 243.82)	220.60 (202.10, 240.79)
Day 5	239.99 (211.30; 272.58)	219.73 (190.21; 253.83)	2.05 (1.56; 2.67)
**Total NNAL**^a^**(pg/mg creat)**			
Baseline	111.01 (95.44; 129.13)	105.05 (84.10; 131.21)	119.40 (94.97; 150.10)
Day 5	49.65 (42.47; 58.05)	107.04 (85.90; 133.37)	41.51 (31.76; 54.26)
**Total NNN**^a^**(pg/mg creat)**			
Baseline	4.81 (3.99; 5.78)	4.34 (3.56; 5.28)	5.14 (3.93; 6.70)
Day 5	1.55 (1.17; 2.05)	5.99 (4.94; 7.26)	0.16 (0.14; 0.19)
**COHb**^a^, ^c^**(%)**			
Baseline	4.65 (4.29; 5.04)	4.68 (4.22; 5.18)	4.96 (4.51; 5.46)
Day 5	1.06 (1.03; 1.08)	4.51 (4.05; 5.01)	0.99 (0.95; 1.03)
**MHBMA**^a^**(pg/mg creat)**			
Baseline	1888.27 (1542.95; 2310.86)	2317.31 (1861.41; 2884.85)	1699.39 (1200.16; 2406.29)
Day 5	192.93 (174.90; 212.83)	2399.40 (1884.60; 3054.83)	163.17 (138.41; 192.36)
**3-HPMA**^a^**(ng/mg creat)**			
Baseline	841.84 (745.88; 950.14)	799.37 (693.71; 921.13)	861.88 (744.98; 997.12)
Day 5	402.26 (366.55; 441.45)	931.01 (825.73; 1049.72)	245.69 (226.15; 266.91)
**S-PMA**^a^**(pg/mg creat)**			
Baseline	2394.03 (1982.83; 2890.50)	2765.20 (2227.38; 3432.88)	2153.82 (1595.57; 2907.38)
Day 5	164.45 (144.45; 187.22)	2922.81 (2362.80; 3615.54)	143.70 (122.15; 169.04)
**Total 1-OHP**^a^**(pg/mg creat)**			
Baseline	217.69 (197.88; 239.48)	218.41 (196.28; 243.03)	223.95 (198.89; 252.17)
Day 5^a^	81.22 (74.82; 88.16)	182.85 (161.24; 207.37)	85.13 (75.37; 96.15)
**4-ABP**^a^**(pg/mg creat)**			
Baseline	13.25 (11.85; 14.81)	13.11 (11.33; 15.16)	13.53 (11.71; 15.63)
Day 5	1.9 (1.70; 2.12)	12.58 (11.03; 14.34)	1.60 (1.40; 1.83)
**1-NA**^a^**(pg/mg creat)**			
Baseline	73.83 (66.48; 81.99)	77.84 (66.32; 91.36)	77.31 (67.76; 88.21)
Day 5	3.30 (2.89; 3.78)	89.37 (77.81; 102.64)	2.56 (2.25; 2.90)
**2-NA**^a^**(pg/mg creat)**			
Baseline	24.54 (22.12; 27.22)	24.14 (21.18; 27.51)	24.59 (21.52; 28.10)
Day 5	2.96 (2.67; 3.28)	25.32 (22.27; 28.79)	2.52 (2.23; 2.84)
**o-tol**^a^**(pg/mg creat)**			
Baseline	135.20 (122.75; 148.90)	131.32 (115.72; 149.01)	132.49 (114.75; 152.97)
Day 5	51.15 (46.10; 56.75)	121.16 (105.07; 139.71)	41.64 (36.74; 47.18)
**CEMA**^a^**(ng/mg creat)**			
Baseline	98.03 (85.10; 112.92)	98.46 (83.81; 115.67)	103.51 (89.35; 119.91)
Day 5	13.18 (11.37; 15.27)	99.48 (85.79; 115.35)	12.6 (10.12; 15.70)
**HEMA**^a^**(pg/mg creat)**			
Baseline	4161.66 (3409.70; 5079.44)	4718.48 (3582.18; 6215.23)	4114.78 (3127.42; 5413.84)
Day 5	1342.40 (1140.44; 1580.12)	4504.00 (3506.73; 5784.88)	1248.27 (980.62; 1588.98)
**3-HMPMA**^a^**(ng/mg creat)**			
Baseline	479.34 (435.40; 527.72)	460.52 (407.39; 520.59)	443.71 (382.03; 515.35)
Day 5	86.65 (80.31; 93.49)	376.78 (329.54; 430.80)	63.25 (57.79; 69.22)
**Total-3-OH-B[a]P**^a^**(fg/mg creat)**			
Baseline	161.17 (142.28; 182.57)	149.47 (128.96; 173.23)	169.58 (147.04; 195.57)
Day 5	37.07 (33.25; 41.32)	130.29 (110.17; 154.07)	33.64 (28.84; 39.24)

Abbreviations: NC=not calculated. CC=conventional cigarette group; SA=smoking abstinence group; THS=Tobacco Heating System 2.2 group.

a Geometric means and 95% confidence intervals are provided in the table. All urinary biomarkers of exposure were expressed as concentrations adjusted to creatinine.

b Weighted average concentration over 24 h (Cavg).

c Measured between 8:00 PM and 10:00 PM.

**Table 2:** Biomarker of exposure, % change from baseline.

Biomarker	THS	CC	SA
	*N*=80	*N*=41	*N*=39
**NEQ (mg/g creat)**			
Change^a^	22.95 (13.92; 31.98)	14.78 (7.04; 22.53)	−98.25 (−98.67; −97.83)
**Nicotine**^b^^**,**^^c^**(ng/mL)**			
Change^a^	35.98 (19.19; 52.77)	19.68 (1.74; 37.62)	−99.19 (−99.40; −98.98)
**Cotinine**^b^^**,**^^c^**(ng/mL)**			
Change^a^	11.94 (4.05; 19.84)	−0.31 (−8.24; 7.63)	−98.83 (−99.10;-98.56)
**Total NNAL (pg/mg creat)**			
Change^a^	-53.98 (−56.69; −51.27)	3.85 (−2.84; 10.54)	−63.95 (−66.98; −60.92)
**Total NNN (pg/mg creat)**			
Change^a^	−69.75 (−53.26;-82.75)#	29.93 (12.84; 74.96)	−97.04 (−94.06; −98.46) #
**COHb**^c^**(%)**			
Change^a^	−76.20 (−78.11; −74.29)	−1.16 (−8.31; 5.99)	−79.24 (−81.09; −77.40)
**MHBMA (pg/mg creat)**			
Change^a^	−84.98 (−88.32; −81.63)	6.89 (−1.37; 15.15)	−79.56 (−88.33; −70.79)
**3-HPMA (ng/mg creat)**			
Change^a^	-49.68 (−54.32; −45.04)	20.28 (10.41; 30.14)	−69.45 (−73.27; −65.63)
**S-PMA (pg/mg creat)**			
Change^a^	−92.03 (−93.20; −90.85)	9.48 (−0.48; 19.45)	−91.52 (−93.72; −89.33)
**Total 1-OHP (pg/mg creat)**			
Change^a^	−60.17 (−63.70; -56.65)	−13.52 (−21.11; −5.92)	−59.94 (−64.26; −55.62)
**4-ABP (pg/mg creat)**			
Change^a^	−82.12 (−85.59; −78.64)	−1.66 (−8.59; 5.26)	−86.99 (−88.87; −85.12)
**1-NA (pg/mg creat)**			
Change^a^	−94.16 (-95.49; −92.82)	19.17 (9.08; 29.26)	−96.41 (−96.89; −95.93)
**2-NA (pg/mg creat)**			
Change^a^	−85.39 (−87.95; −82.82)	7.19 (0.41; 13.97)	−88.93 (−90.38; −87.47)
**o-tol (pg/mg creat)**			
Change^a^	-50.96 (−61.87; −40.05)	−3.08 (−12.17; 6.01)	−64.96 (−70.90; −59.01)
**CEMA (ng/mg creat)**			
Change^a^	-86.10 (−87.04; −85.17)	4.21 (−4.18; 12.61)	−86.74 (−88.30; −85.17)
**HEMA (pg/mg creat)**			
Change^a^	-60.71 (−68.31; −53.11)	0.74 (−9.86; 11.34)	−64.48 (−71.29; −57.66)
**HMPMA (ng/mg creat)**			
Change^a^	-80.58 (−82.48; −78.68)	−14.53 (−22.49; −6.57)	−84.39 (−86.58; −82.20)
**Total-3-OH-B[a]P (fg/mg creat)**			
Change^a^	−71.43 (−76.65; −66.21)	−8.92 (−17.00; −0.84)	−77.24 (−81.14; −73.34)

Abbreviations: NC=not calculated. CC=conventional cigarette use group; SA=smoking abstinence group; THS=Tobacco Heating System 2.2 use group

a Change: Day 5 % change from baseline; arithmetic mean percent change, and 95% confidence interval. Values are derived from concentratiosn adjusted to creatinoen for urinary biomarkers of exposure.

b derived from the weighted average concentration over 24 h.

c Measured between 8:00 PM and 10:00 PM. # for total NNN, the change from Baseline was calculated form the median value.

**Table 3:** Urinary biomarkers of exposure expressed as quantity excreted, ratios of THS relative to CC.

**Biomarkers**	**Ratio**^a^**(% and CI)**
	**THS (N=81)/ CC (N=41)**
**NEQ (mg/g creat)**	97.98 (80.98, 118.54)
**Total NNAL (pg/mg creat)**	42.79 (37.40; 48.95)
**Total NNN (pg/mg creat)**	23.45 (17.12, 32.12)
**MHBMA (pg/mg creat)**	8.17 (6.70; 9.96)
**3-HPMA (ng/mg creat)**	40.49 (35.84; 45.74)
**S-PMA (pg/mg creat)**	5.87 (5.03; 6.85)
**Total 1-OHP (pg/mg creat)**	43.43 (37.67; 50.06)
**4-ABP (pg/mg creat)**	14.55 (12.51; 16.92)
**1-NA (pg/mg creat)**	3.66 (3.06; 4.37)
**2-NA (pg/mg creat)**	11.22 (9.75; 12.91)
**o-tol (pg/mg creat)**	40.38 (34.92, 46.68)
**CEMA (ng/mg creat)**	12.96 (11.15, 15.06)
**HEMA (pg/mg creat)**	31.37 (26.51, 37.11)
**3- HMPMA (ng/mg creat)**	21.85 (18.83; 25.36)
**Total 3-OH-B[a]P (fg/mg creat)**	26.62 (22.33, 31.74)

a Ratio: Geometric least squares mean ratio (%) and confidence intervals from an ANCOVA model conducted on log-transformed Day 5 values (urinary biomarker of exposure expressed as quantity) with log-transformed baseline value, study arm, sex and CC consumption reported at screening as fixed effect factors (THS/CC) on Day 5. Abbreviations: NC=not calculated. CC=conventional cigarette group; CI: Confidence interval; THS=Tobacco Heating System 2.2 group.
